# Two-dimensional dynamic multileaf collimator (2DDMLC) technique for improved treatment quality in intensity modulated radiation therapy

**DOI:** 10.1038/s41598-025-01002-5

**Published:** 2025-05-19

**Authors:** Hyojun Park, Chul Hee Min, So-Yeon Park, Jung-In Kim

**Affiliations:** 1 Department of Radiation Oncology, Seoul National University Hospital, Seoul, 03080 Korea; 2 Institute of Radiation Medicine, Seoul National University Medical Research Center, Seoul, 03082 Korea; 3 Department of Radiation Convergence Engineering, Yonsei University, Wonju, 26493 Korea; 4 Department of Radiation Oncology, Veterans Health Service Medical Center, Seoul, 05368 Korea; 5 Biomedical Research Institute, Seoul National University Hospital, Seoul, 03082 Korea

**Keywords:** Two-dimensional dynamic multileaf collimator, Intensity modulated radiotherapy, Field optimization, Normal tissue sparing, Radiotherapy, Radiotherapy

## Abstract

This study aims to evaluate feasibility of the two-dimensional dynamic MLC (2DDMLC) technique through analytical and experimental investigations, focusing on its potential to improve intensity-modulated radiation therapy (IMRT). The leaf motion calculator (LMC), which calculates the leaf motion of the MLC during the treatment, was developed to obtain the MLC sequence including the bank’s movement and anticipate the actual fluence delivery. The effect of the y-axis MLC motion was evaluated by calculating the actual fluence distributions from the optimal fluence maps. The actual fluence maps were compared to those of the conventional MLC. The conformity index (CI) and average difference from the optimal fluence map were calculated. Subsequently, prototype of the 2DDMLC was manufactured by using the Varian Millennium 120^TM^ MLC to measure dose distributions according to presence of MLC’s y-axis movement. The dose distributions were compared to their optimal fluence maps according to different MLCs in terms of dose sparing to the volume outside the tumor. The beam fluences produced by the 2DDMLC technique showed better conformity to their optimal fluence distributions than the conventional MLC. The 2DDMLC technique enhanced the beam conformity compared to the conventional MLC, as indicated by a CI closer to 1, indicating better target conformity. In addition, difference between the actual and the optimal fluence maps was reduced by a factor of 2. The dose distributions were improved by the 2DDMLC as the irradiation area outside the target region was reduced by 50% in maximum. For example, the normal tissue irradiation area was reduced by 49% and 24% in the lung and head & neck cases, respectively. The 2DDMLC technique can contribute enhanced normal tissue sparing with less probability of side effects. As extension of this study, the suggested device can be employed to the volumetric modulation arc treatment.

## Introduction

Multileaf collimator (MLC) is fundamental in modern radiotherapy using high-energy X-ray, playing a crucial role in shaping the radiation beam to be conformal to the tumor volume. It produces complex dose distributions conformal to tumor volume in the patient body. Spatial resolution of the dose distributions is determined by the leaf width of the MLC. The leaf width is an important parameter, which affects treatment quality, such as dose conformity and normal tissue sparing. Several studies investigated the effects of the leaf width on the treatment outcomes of the intensity modulated radiotherapy (IMRT) and volumetric modulated arc therapy (VMAT)^1–10^. These studies consistently demonstrated improved dose conformity and normal tissue sparing with finer MLCs leaf thicknesses. However, there is a limitation on reducing the leaf width to improve treatment result. It was reported that the ideal thickness of the MLC leaf was reported from 1.5 to 1.8 mm^11^. There is no benefit on thinner MLC than the ideal thickness and treatment results can even be worsened due to the leakage dose^9,10,12,13^. Commercialized MLCs already accomplished the optimal thickness range, such as the Varian high-definition MLC (HD-MLC). In addition, technological constraints limit further advances. Space constraint inside the gantry head restricts the number of available motors and controllers to move each leaf. Thinner MLCs also reduce the maximum achievable field size compared to thicker counterparts.

There have been efforts to enhance dose conformity without reducing the leaf width, including advanced MLC systems. The VERO dynamic MLC tracking system was developed by BrainLab (AG, Munich, Germany) and MHI (Mitsubishi Heavy Industries, Minato, Tokyo, Japan)^14^. This system tracks the tumor volume in real-time during the treatment to optimize the beam field. The dual-layer MLC, which stacks and staggers two independent MLC layers, has been introduced^15–19^. This MLC is applied to Halcyon^TM^ (Varian Medical Systems, Palo Alto, USA) and MRIdian (ViewRay Inc, Mountain View, USA). However, the VERO MLC requires acquisition of kilo-voltage (KV) fluoroscopy during the treatment to track the tumor volume, which delivers additional dose to the patient. The resolution of the dose distribution is not changed. Meanwhile, the dual-layer MLC increases uncertainty of leaf positioning with increased number of the leaf pair despite the improvement of the dose resolution. Additionally, both systems face limitations regarding simplicity of installation and space constraint within the gantry.

**Fig. 1:**
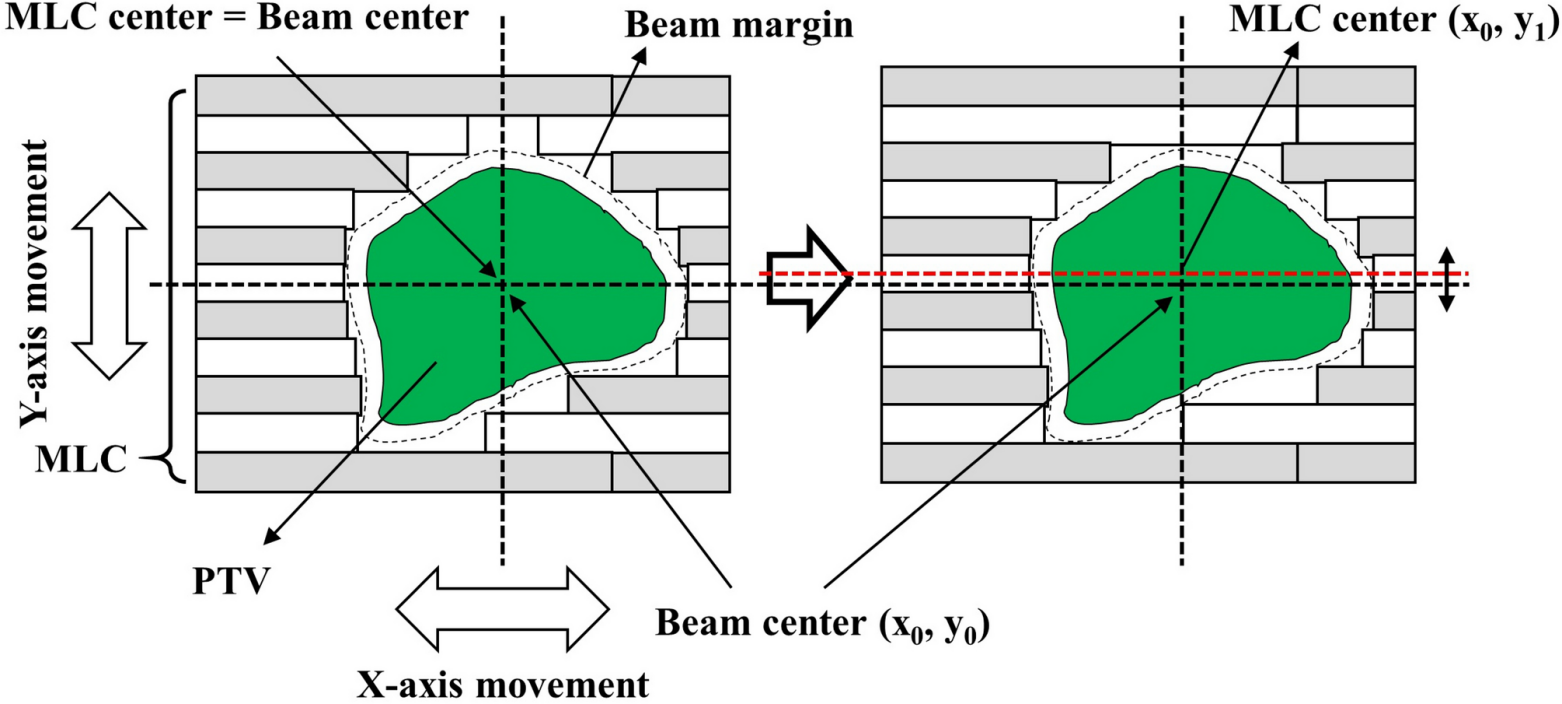
Concept of the 2DDMLC technique with definition of the x- and y-axis movement. Note that, the beam aperture is more conformal after optimizing MLC’s y-axis coordinate from y_0_ to y_1_.

As one of the alternative approaches, we have proposed a technique, so-called as two-dimensional dynamic MLC (2DDMLC), which moves along both x- and y-axis (Fig. 1)^20^. This technique moves the MLC bank along the y-axis while the leaf travels along the x-axis. The MLC optimizes its opening by adjusting the y-axis position throughout comparison of MLC openings at different y-axis positions. This minimizes beam delivery to the normal tissues. The 2DDMLC has advantages on simplicity of installation compared to the VERO and dual-layer MLC. The MLC technique showed its clinical feasibility by enhancing normal tissue sparing without compromising the tumor dose. However, the study mainly focused on the Monte Carlo (MC)-based proof-of-concept using dynamic conformal arc (DCA) therapy. Experimental validation is required for concrete and realistic validation of clinical availability of the 2DDMLC. The beam delivery should be analyzed based on the measurement using the actual machine. Moreover, considerations for different tumor sites and treatment techniques are required.

This study aimed to experimentally validate the 2DDMLC technique in terms of its clinical feasibility, and investigate its potential to improve radiation treatment quality. A prototype of the 2DDMLC was developed by using a commercial MLC and a linear actuator for the MLC’s bank movement. An algorithm to calculate the leaf sequence for changing bank position was developed since current leaf motion calculators (LMCs) do not perform the optimization of the bank position. Subsequently, dose distribution was measured to evaluate the clinical effectiveness of the 2DDMLC by using both an actual medical linear accelerator (LINAC) and the 2DDMLC prototype.

## Results

### Validation of the LMC algorithm

**Fig. 2:**
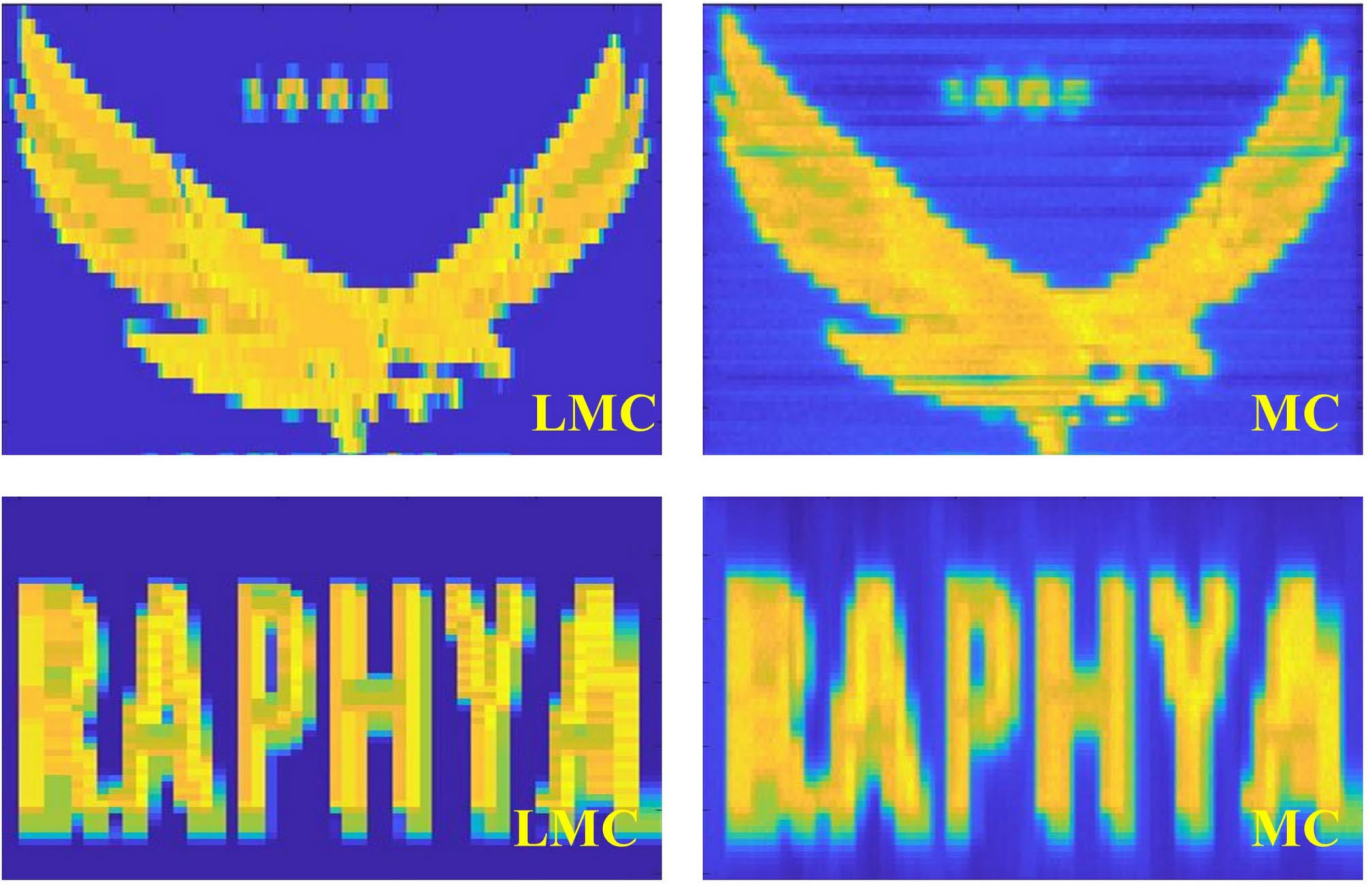
Fluence distributions obtained from the LMC (left) and the Geant4 MC simulation (right).

Figure 2 shows the actual fluence maps obtained from the LMC and the GEometry ANd Tracking 4 (Geant4) simulation. The fluence maps obtained from the simulation was slightly more blurred than those calculated by the LMC. Each subfield comprising the fluence distribution was very small as its width was less than 1 mm. The uniformity of the fluence in each subfield was not preserved in the simulation, whereas the LMC assumed the ideal uniform beam delivery for each subfield. Additionally, the fluence in the region outside the planned irradiation area was higher in the simulation results compared to those from the LMC as the actual fluence map of the LMC did not consider the leakage. Despite these differences, the shape of the fluence distribution was almost identical between the LMC and the simulation. Since the leakage affects the fluence distribution as a constant factor, the primary consideration for evaluating the LMC was the accuracy of the fluence delivery to achieve the exact shape of the optimal fluence. Therefore, the LMC showed its availability on calculating the leaf sequence and the actual fluence map as well. The algorithm accounts for the y-axis position of the MLC when it calculates the leaf sequence and the actual fluence map. This make it suitable for the 2DDMLC technique as it includes the optimization of the beam fields by comparing the actual fluence maps according to the different y-axis positions.

### Comparison of the beam delivery by the conventional MLC and 2DDMLC

**Fig. 3:**
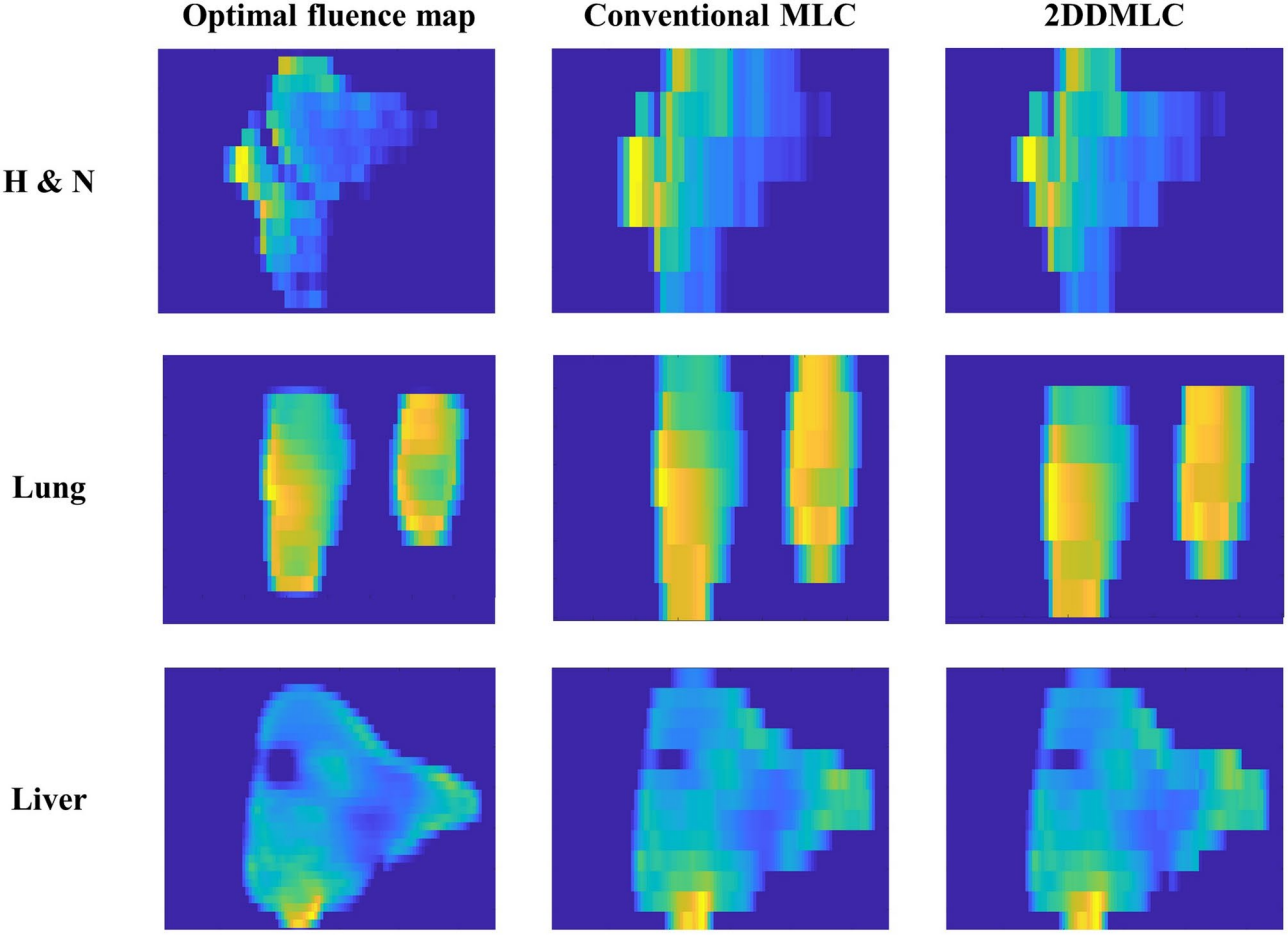
Representative fluence distributions by the conventional MLC and the 2DDMLC. Optimal fluence maps (left), actual fluence maps of conventional MLC (center) and the 2DDMLC (right) are illustrated. Height of the target volume was 2.8, 2.8, and 6 cm, for H&N, lung and liver cases, respectively.

The actual fluence maps calculated using the LMC were compared between the conventional and 2DDMLC techniques. Figure 3 shows the optimal fluence maps and the actual fluence maps with the conventional and 2DDMLC techniques for representative cases of head and neck (H&N), lung, and liver cancers. The difference between the optimal and actual fluence maps were smaller with the 2DDMLC technique compared to that with the conventional MLC. The difference reduced from 19.2 to 13.9% and from 15.4 to 8.5% in the H&N and lung cases, respectively. Meanwhile, the actual fluence maps showed similar difference from the optimal fluence map in the case of liver cancer; that was, the differences were 11.0% and 10.8% with the conventional and 2DDMLC techniques, respectively. These results demonstrate that the 2DDMLC technique delivered the beam fluence closer to the optimal fluence map compared to the conventional MLC. Smaller region was irradiated in the out-of-field area with the 2DDMLC. In the case of liver cancer, effect of the 2DDMLC was insignificant due to size of the target volume (5-6 cm or larger). The 2DDMLC technique provides dosimetric advantages by minimizing the irradiation in the region outside the target volume, especially at the target border. This is mainly achieved within the distance of the leaf width, which was 5 mm for the MLC used in this study. Table 1 shows the mean intensity of the actual fluence maps and the average conformity indices for different cancers. The mean intensity was identical in all cases between the MLC techniques. The conformity index (CI), which indicates better conformity with a value closer to 1, shows enhanced beam conformity with the 2DDMLC technique compared to the conventional technique. In the H&N case, the CI was 1.26 and 1.17 with the conventional- and 2DDMLC techniques, respectively. The CIs of the lung case were 1.27 and 1.17 while the 2DDMLC technique shows better conformity. This indicates the 2DDMLC technique can reduce unnecessary beam delivery to the normal tissues while it maintains the dose distribution in the target volume.

**Table 1:** Mean intensity and conformity index of the fluence maps for different tumor sites.

Tumor site	Index	Conventional MLC	2DDMLC	p
Lung	Mean Intensity	0.13 ± 0.03	0.13 ± 0.03	–
	CI	1.27 ± 0.08	1.17 ± 0.05	$< 0.01$
Esophagus	Mean Intensity	0.16 ± 0.02	0.16 ± 0.02	–
	CI	1.17 ± 0.03	1.11 ± 0.03	$< 0.01$
H&N	Mean Intensity	0.14 ± 0.05	0.14 ± 0.05	–
	CI	1.26 ± 0.10	1.17 ± 0.06	$< 0.01$
Breast	Mean Intensity	0.11 ± 0.02	0.11 ± 0.02	–
	CI	1.10 ± 0.02	1.06 ± 0.02	$< 0.01$
Abdomen	Mean Intensity	0.22 ± 0.04	0.22 ± 0.04	–
	CI	1.20 ± 0.05	1.11 ± 0.03	$< 0.01$
Mediastinum	Mean Intensity	0.16 ± 0.03	0.16 ± 0.03	–
	CI	1.14 ± 0.01	1.09 ± 0.01	$< 0.01$
Liver	Mean Intensity	0.14 ± 0.01	0.14 ± 0.01	–
	CI	1.13 ± 0.01	1.09 ± 0.01	$< 0.01$
Others	Mean Intensity	0.15 ± 0.02	0.15 ± 0.02	–
	CI	1.08 ± 0.02	1.05 ± 0.02	$< 0.01$

### Comparison of dose distributions between the conventional MLC and 2DDMLC

**Fig. 4:**
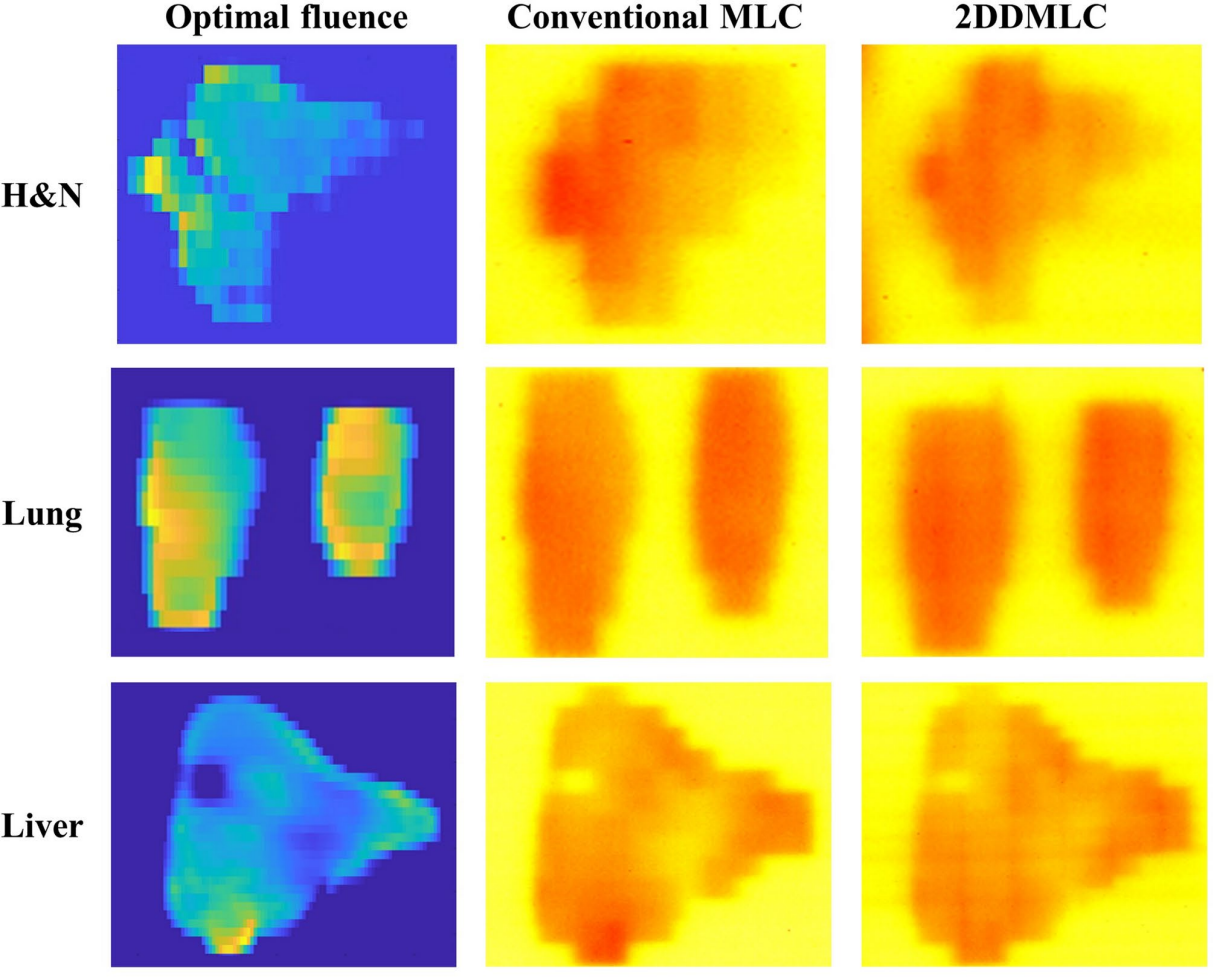
Dose Distributions according to different MLC operation techniques measured with the commercialized device. Figures on left-side are the optimal fluence maps. Figures at center and right-side are obtained by using the conventional MLC and 2DDMLC techniques, respectively.

**Fig. 5:**
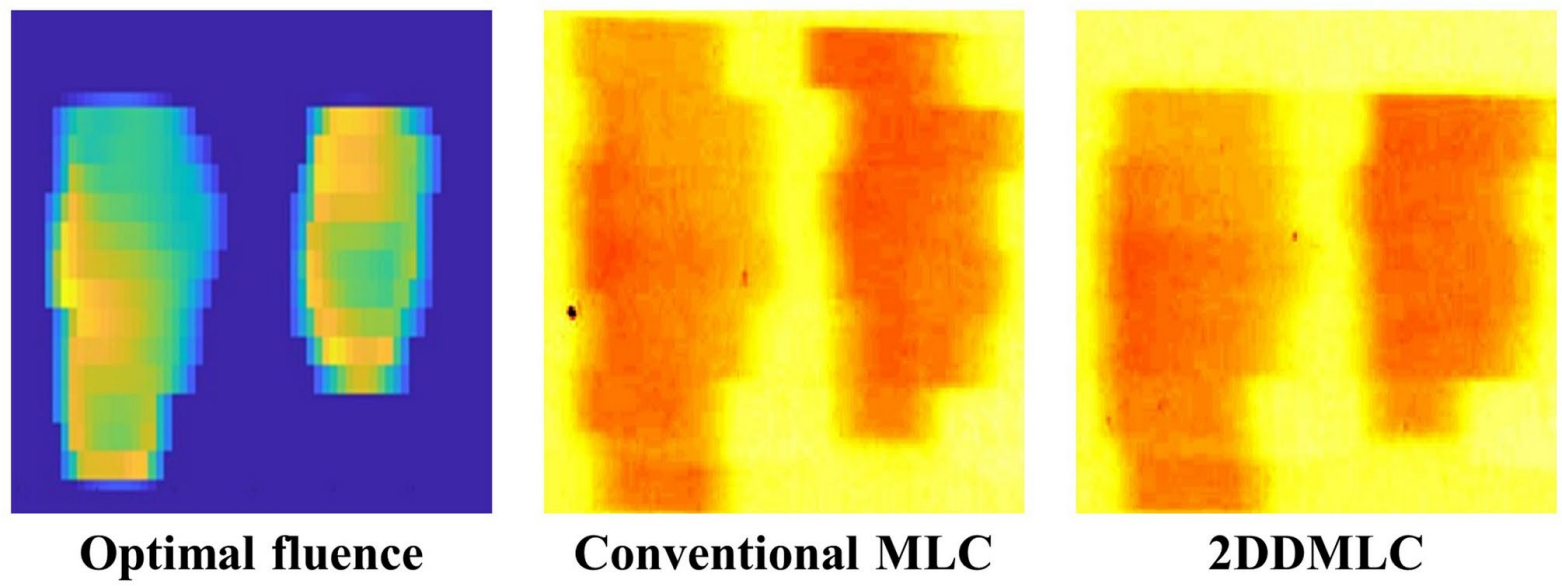
Dose distribution obtained by using the conventional MLC (center) and the 2DDMLC prototype (right) based on the optimal fluence map of lung case (left).

The results of the film measurements with the commercial LINAC are shown in Fig. 4. The dose distributions were similar to the actual fluence maps calculated using the LMC. This indicates that the accuracy of the LMC’s actual fluence map is acceptable, as validated through the MC investigation. The dose distributions show similar dose delivery to the area outside the optimal fluence distribution, as calculated by the LMC. Normal tissue sparing was enhanced in a manner similar to the calculation with the LMC. Sizes of the unwanted irradiation areas were calculated by comparing the optimal fluence map and the dose distributions of the different MLC techniques. The unwanted irradiation area was reduced by 24% and 49% in the H&N and lung cases, respectively. This reduction mainly occurred at upper side of the dose distribution, which suggests that the 2DDMLC moved up and closed the leaf pair at top of the optimal fluence map for both cases. As calculated by the LMC, the dose distributions were also similar in the case of liver cancer due to the size of the optimal fluence distribution being larger than the distance of the MLC’s y-axis movement and the leaf width. Figure 5 shows the dose distribution measured by using the 2DDMLC prototype. The dose distributions were similar to those measured with the commercial LINAC. The dose to the outside area of the optimal fluence distribution was reduced by 47% with the 2DDMLC technique. The compartments of the 2DDMLC prototype were not fully compatible to each other, since they were separately produced by different manufacturers. The dose distributions were partially different from those measured with the commercial machine. Additionally, the MLC did not move precisely as did the commercial device due to the functional limitation of the separately manufactured MLC controller. This limitation should be addressed with a controller of enhanced performance.

## Discussion

Validation of the 2DDMLC technique was carried out through both analytical and experimental investigations. The LMC algorithm was developed for the 2DDMLC technique. It was validated by comparing calculated actual fluence distributions to those obtained from the MC simulations. The fluence distributions were compared between the conventional and 2DDMLC techniques. Subsequently, experimental validation of the 2DDMLC technique was carried out by using the conventional device and prototype of the 2DDMLC. The fluence and dose distributions showed that the 2DDMLC technique notably reduced irradiation region outside of the planned target area compared to the conventional technique.

The results demonstrate the potential of the 2DDMLC to enhance treatment quality of the IMRT. The LMC was developed to calculate the MLC sequences with the consideration of the MLC’s y-axis position. The dosimetric advantage of the 2DDMLC technique was evaluated with the results of the experiments using the commercial LINAC and the 2DDMLC prototype. The 2DDMLC showed improved dose conformity, which is expected to enhance normal tissue sparing during the treatment.

The actual fluence maps of the 2DDMLC technique showed less irradiation to area outside the target volume, improved beam conformity, and higher accordance with the optimal fluence map compared to those of the conventional technique. Consistent with the LMC calculations, the results of the film measurements demonstrated a reduction of unwanted irradiation area outside the target volume when using the 2DDMLC technique instead of the conventional MLC. The effect of the 2DDMLC technique on the beam conformity was less observed with large targets. This is due to the ratio of the leaf width to the vertical length of the target as the distance of the MLC y-axis movement is defined as half of the leaf width. This implies that the dosimetric effect is confined to 2.5-mm regions at the superior and inferior margins of the tumor. Therefore, the 2DDMLC technique is expected to be more effective in the treatment of small-size cancers or the treatments composed of several small-sized subfields. It can also be applied to the stereotactic ablative radiotherapy (SABR), which requires higher dose conformity and concentration of a large amount of dose in a small treatment area as well. This study can be extended to assess the application of the 2DDMLC technique in patients with different tumor sites. A dosimetric analysis considering parameters such as tumor size, location, and shape could provide valuable data to prove 2DDMLC’s clinical feasibility. This requires an evaluation of dose distribution in the patient body and MC simulation could be one of the best approaches. Since the previous study was limited to brain cancer cases^20^, further study will be focus on investigating the application of the 2DDMLC in various tumor types.

The 2DDMLC prototype was made by combining the linear actuator with the MLC currently used in the clinic. It was sufficient to evaluate the clinical availability of the 2DDMLC. However, for clinical application of the 2DDMLC as the actual treatment device, the 2DDMLC should be fully compatible with the LINAC as well as its components should also be compatible with each other. The 2DDMLC prototype cannot vary the leaf travel speed according to dose rate or beam on/off status of the LINAC. The MLC should communicate with the LINAC to automatically trigger start/stop of the leaf travel by considering the machine status. It is required to embed device that enables the communication between the MLC and the LINAC. The linear actuator for the MLC’s y-axis movement requires higher precision. It should be more powerful to support substantial weight of the MLC bank while ensuring precise movement within a 0.25-mm step. Alternatively, developing a lighter MLC dedicated for the 2DDMLC technique can be reduce mechanical demands. These improvements can increase accuracy of the MLC positioning and be implemented during the manufacturing process of the 2DDMLC test device for clinical application. Further investigations and efforts are required to address these challenges and to enable the 2DDMLC to be used in the actual treatment.

In this study, the 2DDMLC technique defined the optimal y-axis position of the MLC for each segments of the optimal fluence maps while the fluence maps were divided into 8 segments. Increasing the number of segmentation can improve dose conformity. However, this also compromises the optimization speed of the MLC and highlights mechanical limitation of the MLC’s y-axis movement. Considering the speed of the leaf, the y-axis motor of the MLC should arrive at the optimized position within a very short time and with very high precision. They should be taken into account to ensure more fluent movement of the 2DDMLC and to minimize the risk of treatment failure. These factors will be addressed in further studies focused on developing the 2DDMLC device for clinical application.

Currently, the effectiveness of the 2DDMLC was evaluated in terms of the conformity index and the irradiated region outside the originally planned area, as this study mainly focused on improving field shaping of the MLC. In addition, parameters such as homogeneity index could be used to evaluate 3D dose distribution in tumor. Our results refer to beam fluence in the beam’s eye view of each gantry angle. Therefore, these factors are less suitable for analyzing the findings of current study. Further investigations will be carried out on the analysis of dose distribution in the patient body with the IMRT by using computational methods such as the MC simulations. In this process, other parameters can be quantified to evaluate dosimetric advantages of the 2DDMLC in IMRT, such as homogeneity index and equivalent uniform dose (EUD) to the target or OARs. Our previous study was similarly carried out the MC simulations of DCA therapy^20^. However, the study only produced the MLC aperture based on the projection of the PTV contour from each gantry angle. Further simulations of IMRT with the 2DDMLC are necessary. Additionally, an application test of the MLC sequence defined by the LMC of the 2DDMLC is required.

The 2DDMLC technique aimed to static IMRT, which consists of several beam fields from different gantry angles. However, the utilization of the VMAT becomes more common than that of the static IMRT. With this circumstance, further study is required to apply the 2DDMLC on the VMAT to expand its applicability. Since the plan optimization is different between the IMRT and VMAT, additional algorithm for the VMAT optimization of the 2DDMLC is required. The LMC was designed to follow the inverse planning method, which requires the optimal fluence map to decide the MLC sequence. For the VMAT application, the algorithm should be compatible with forward planning. In addition, the MLC should be attached to the gantry head during VMAT. Mechanical improvement of the MLC - such as reduction of its weight, enhancing adhesive stability - is necessary. This could be a burden of gantry rotation, leading to a reduction of treatment accuracy. Investigation on the application of the 2DDMLC to the VMAT will be performed with more comprehensive clinical trials. By achieving these mile stones, the 2DDMLC technique is expected to promise improved quality of radiation treatment and treatment outcomes using high energy X-rays.

## Methods

### Development of LMC for the 2DDMLC

The treatment planning system (TPS) determines the optimal beam intensities delivered to each treatment location to maximize dose to tumor volume while minimizing exposure to normal tissues. These beam intensities are defined in the beams’ eye view and the 2D distribution of the optimized beam intensities is called as the optimal fluence map. The LMC calculates the MLC sequence based on the optimal fluence map, taking into account the physical properties of the MLC. It determines position of each leaf at each control point during treatment by modulating speed of the leaf travel to achieve the optimal fluence map. The LMC also calculates the beam intensities modulated by the MLCs movement according to its sequence. The resulting 2D distribution of the beam intensities representing the anticipated beam delivery during the treatment is known as the actual fluence map. This fluence map is used to calculate patient’s dose distribution produced by the treatment plan.

Commercial LINACs restrict the MLC movement to the x-axis, where the bank of the MLC does not move vertically to the leaf motion. Therefore, the LMC in the treatment planning system (TPS) only calculates the leaf travel. An in-house developed LMC was used to determine both the leaf travel in the x-axis and the bank movement in the y-axis during treatment. The x-axis movement of the MLC follows the calculation process of leaf motion implanted in the Eclipse^TM^ TPS. The MLC algorithm was designed to follow the LMCs in inverse planning. The algorithm defined the leaf travel to use sliding window method. The algorithm firstly reads the intensity distributions of the optimal fluence map. These intensity distribution can be converted to monitoring units (MUs), which indicates the beam-on time, by considering the machine’s dose rate. Subsequently, it calculates the MUs assigned for both left and right leaves of the MLC at each beamlet. The MUs refers to the time that the MLC leaf should arrive at the beamlet after beam-on. Since the algorithm uses sliding window method, the calculation of MU also considers the time to move each leaf from point to point. This can be achieved by adding MUs of extra time required to move the leaf to the destination. The optimal fluence map is divided into sectors and the LMC calculates independent y-axis positions of the MLC for each. The maximum distance of the MLC’s y-axis movement is defined as half of the leaf width to avoid repeating the same beam field during the optimization of the beam field.

### Validation of the LMC and feasibility evaluation of the 2DDMLC technique

**Fig. 6:**
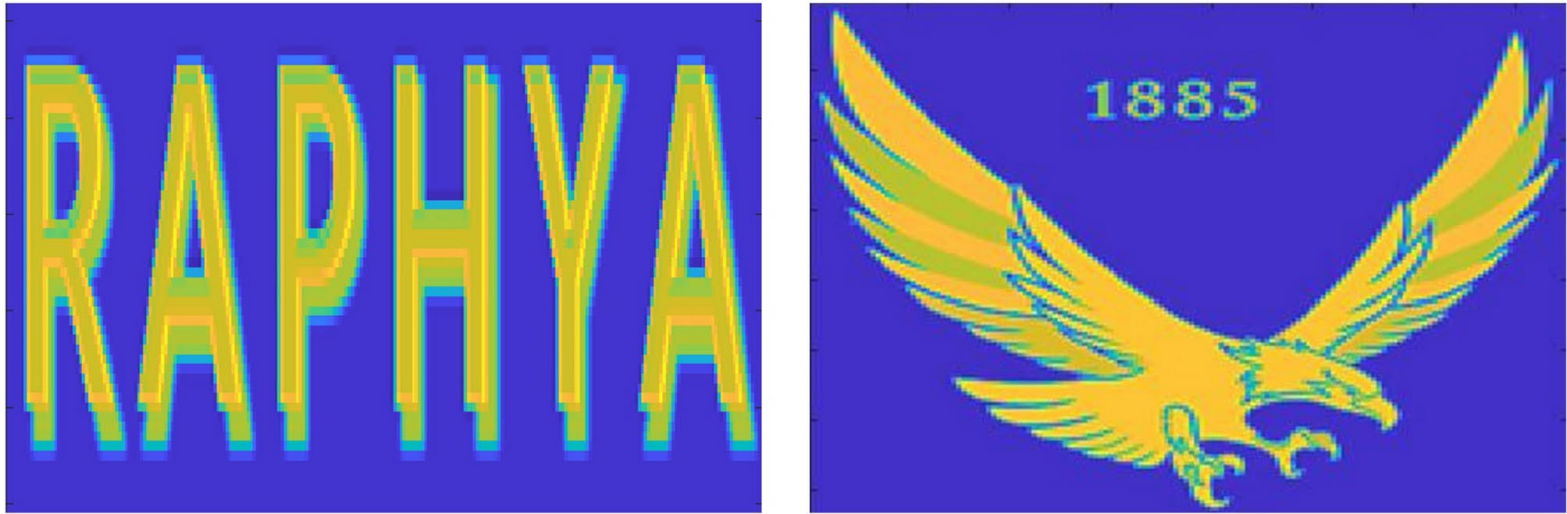
Optimal fluence maps for the validation of the LMC to calculate the leaf sequence of the 2DDMLC. Two different fluence maps were employed - letter (left) and eagle mark with small numbers (right).

The LMC was validated by comparing the actual fluence maps calculated by the LMC to those obtained from the Geant4 simulation including the model of the MLC. Two optimal fluence maps were used in the validation (Fig. 6). Resolution of the fluence map was 1 mm^2^. For the simulation, geometry of the MLC was modeled as described in the previous study^21^. The MLC moved as following its sequence calculated by the LMC to achieve the optimal fluence distribution. The MLC was located at 51 cm from the X-ray source. Field size was 10$\times$10 cm^2^ and the number of generated X-rays was 2$\times$10^7^ for each simulation.

Feasibility of the 2DDMLC technique was evaluated by comparing the actual fluence maps according to presence of the MLC’s y-axis movement. The 2DDMLC was applied to 23 treatment cases given the IMRT for different cancers between 2017 and 2022, encompassing a total of 113 optimal fluence maps with a beamlet size of 1 mm^2^. All of the treatments used sliding window technique to move the MLC leaves. This study was approved by the institutional review board of Seoul National University Hospital (IRB No. 2411-093-1587). The treatment cases were classified into 8 groups in terms of tumor location, which were lung, esophagus, H&N, breast, abdomen, liver, mediastinum, and others. The actual fluence maps were calculated by using the LMC. The optimal fluence map was divided into 8 segments to determine the y-axis position of the MLC. The segmentation allowed for variations in the y-axis position according to different sections of the optimal fluence map, depending on its shape. The y-axis position of the MLC was decided by comparing the beam fields within ±2.5-mm distance of the y-axis MLC translation in 0.25 mm interval. The actual fluence maps were compared between with and without the MLC’s y-axis movement in terms of their conformity and difference from the optimal fluence map. The conformity was evaluated by calculating the factor defined as^22^:

$$\begin{aligned} \mathrm {Conformity\;Index\;(CI)} = \frac{\mathrm {Actual\;irradiation\;area}}{\mathrm {Planned\;irradiation\;area}} \end{aligned}$$

The equation was referred to that for analyzing the dose distribution, which typically considered as one of the dose-volumetric parameters, while the term volume was replaced with size of the irradiation area^22^. The significance of the results was evaluated in terms of the p-values calculated from the paired t-test (*p*< 0.05).

### Experimental validation of the 2DDMLC technique with the conventional LINAC

**Fig. 7:**
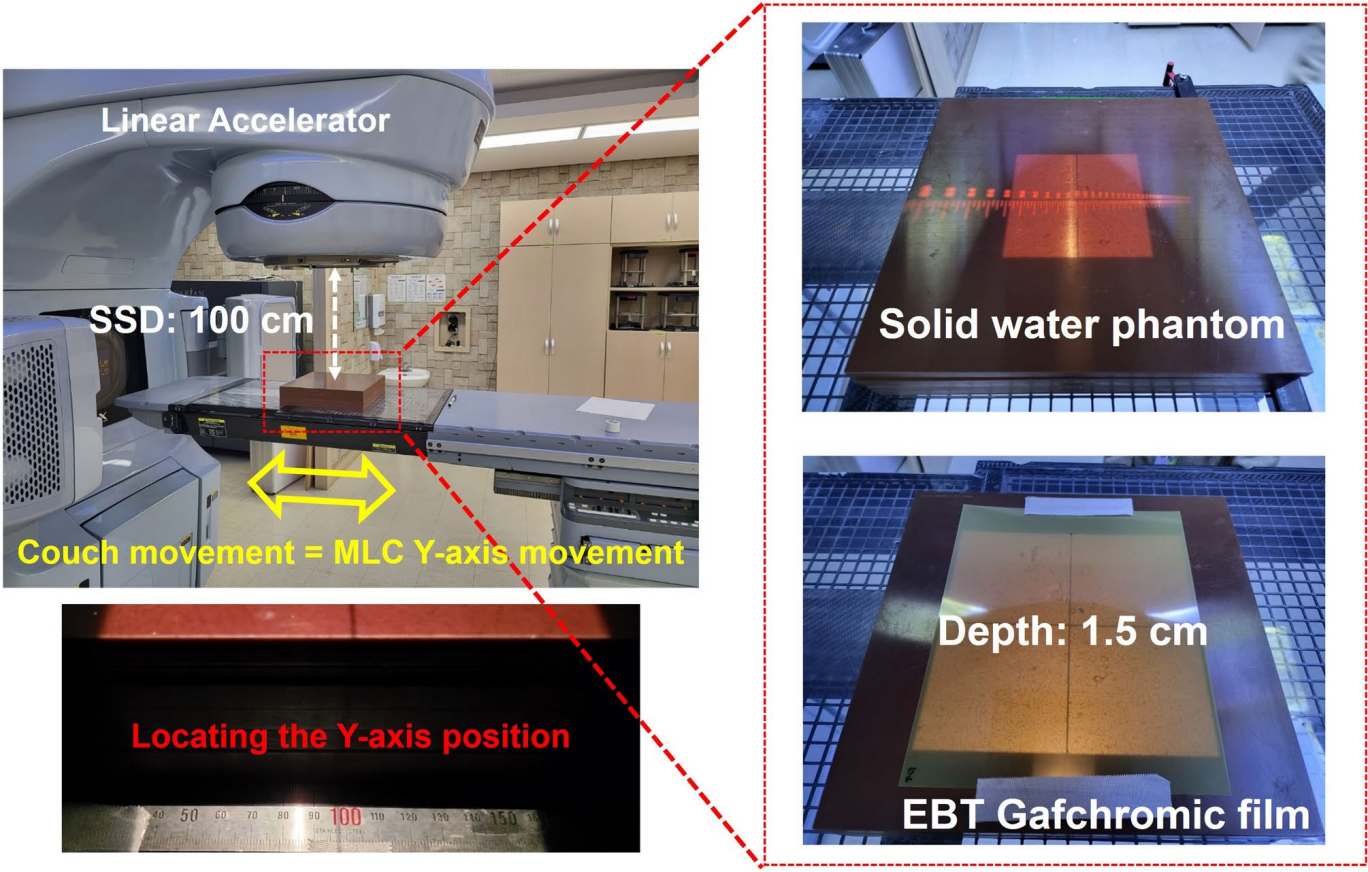
Experiment condition by using the conventional LINAC and MLC to measure dose distributions according to application of the y-axis movement of the 2DDMLC.

**Fig. 8:**
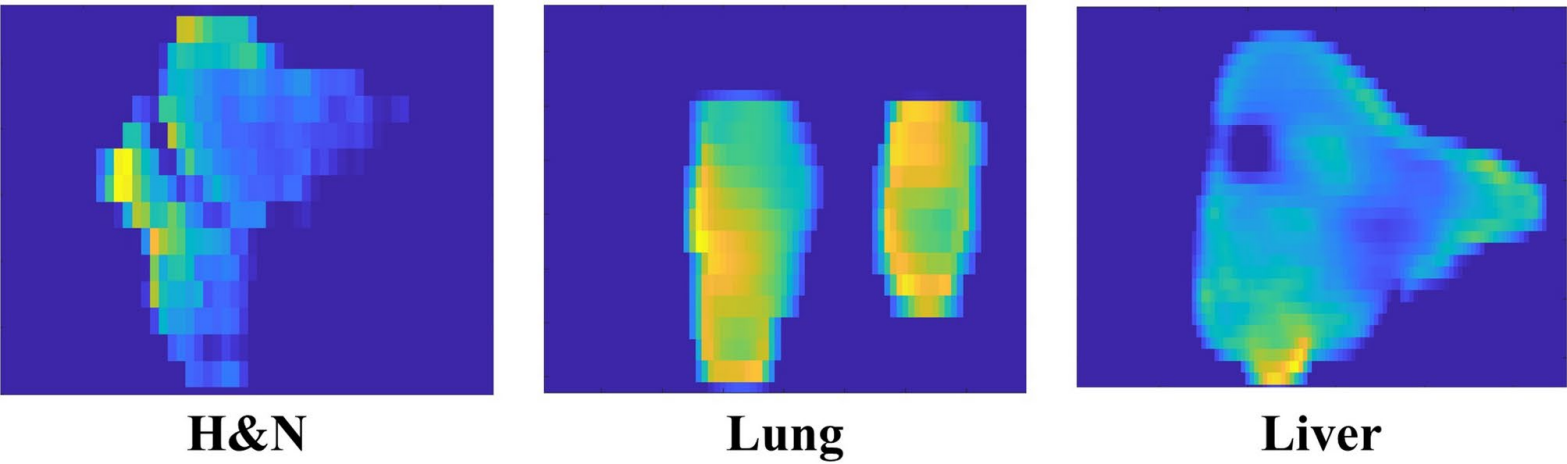
Optimal fluence maps used in the measurement to prove clinical availability of the 2DDMLC..

An experiment was performed to evaluate dosimetric effectiveness of the 2DDMLC technique by using the commercial LINAC (Fig. 7). The dose distributions for different optimal fluence maps were compared between two cases: one with the MLC’s y-axis movement and the other without the y-axis movement. The Varian Clinac iX and Millennium 120^TM^ MLC were used in the experiment. Since commercial LINACs are not able to move the MLC bank, the couch was moved along the longitudinal direction instead. The dose distributions were measured with the EBT Gafchromic film. Measurement depth was 1.5 cm, approximating the depth of maximum dose (d_max_) of 6 MV X-ray, while distance between the X-ray source and the film was 100 cm. The beam was delivered by 500 MU at a dose rate of 600 MU/min. In all cases, the MLC moved in the same way as the sliding window. Figure 8 shows the optimal fluence maps used in the experiment, which were included in the treatment plans for H&N, lung, and liver cancer. The size of the irradiated region outside the optimal fluence map was compared between the 2DDMLC and the conventional MLC techniques. The average difference was calculated between the optimal fluence map and dose distribution. The CI was also assessed using the same equation used to compare the actual fluence maps.

### Experiment with the 2DDMLC prototype

**Fig. 9:**
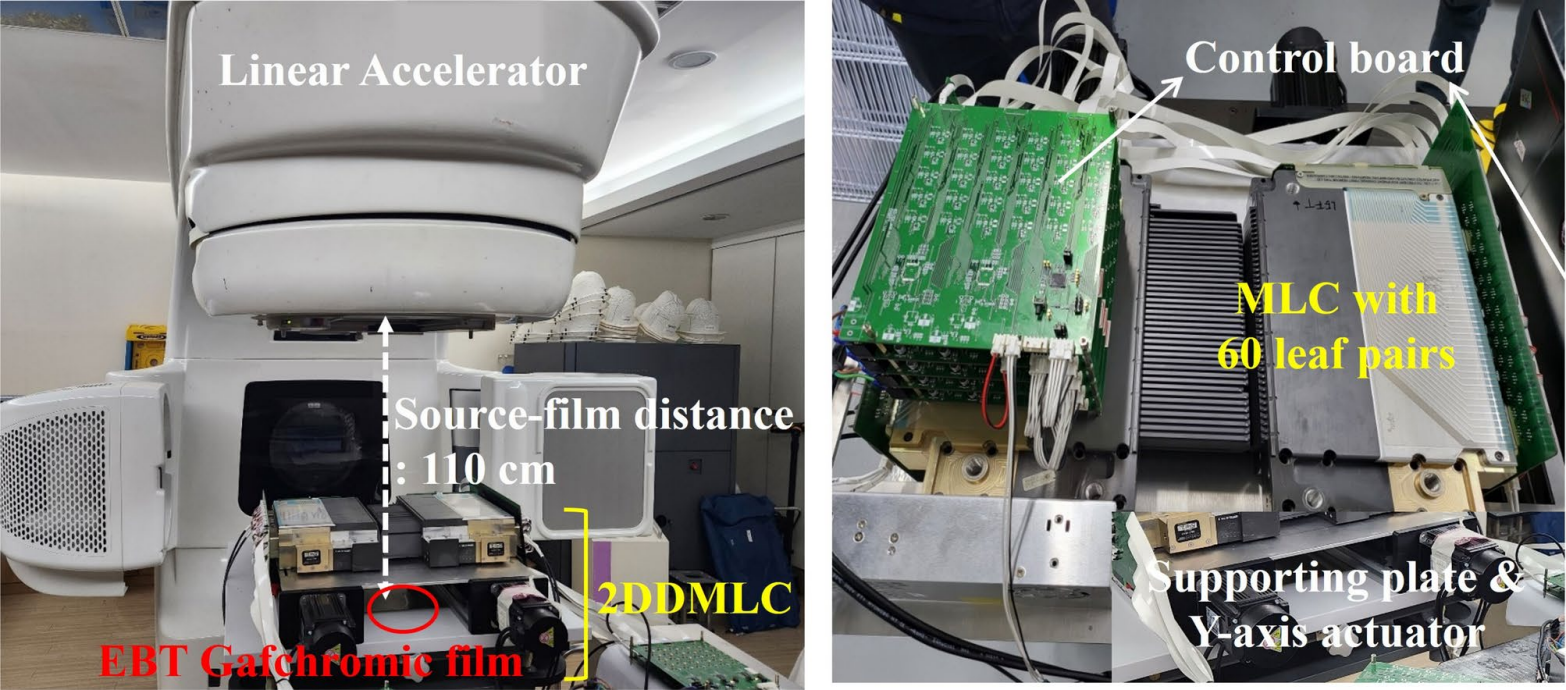
Measurement setup (left) to obtain dose distribution of the 2DDMLC prototype (right).

An experimental validation of the 2DDMLC technique was carried out with the prototype of the MLC (Fig. 9). In the validation, the dose distribution with the 2DDMLC technique was measured and compared to that with the conventional MLC technique; the one with the MLC y-axis movement and the other without the y-axis movement using the same MLC hardware. A prototype of the 2DDMLC is depicted in Fig. 9. It consists of the Varian Millennium 120^TM^ MLC and two linear actuators to move the MLC along the y-axis direction. The MLC has 60 leaf pairs with a leaf width of 5 mm for inner 40 leaves and 10 mm for outer 20 leaves. The actuators support each bank of the MLC and move simultaneously along the vertical direction to the leaf motion. The EBT Gafchromic film was used to measure the dose distribution. Due to weight of the prototype MLC, a water phantom reservoir was used instead of the treatment couch. The distance between the X-ray source and the film was 110 cm since available height of the reservoir is lower than that of the couch. Each measurement involved irradiation of the film with 400 MU of 6 MV X-rays. Similar to the experiment with the conventional device, sliding window technique was applied to the movement of the MLC leaves during beam delivery. The optimal fluence map of the lung tumor was used to generate the MLC sequence since it showed the largest enhancement of dose conformity with the 2DDMLC technique.

## Data Availability

All data generated or analyzed during this study are included in this published article.
